# The Medical Congress

**Published:** 1887-09

**Authors:** 


### THE MEDICAL CONGRESS.

Before this number shall have reached any of its distant subscribers, the International Medical Congress of 1887 will have commenced its sessions. What it will bring forth remains to be seen, but high anticipations of a profitable meeting are indulged in. The Congress in London stimulated an inquiry and encouraged a study that has resulted in a marked advance in dentistry. It is sincerely to be hoped that the coming meeting will be productive of yet greater good, and that its influence will be actively felt for many years to come. While the social aspects of the occasion should by no means be forgotten or neglected, it should steadily be borne in mind by those having charge of the affair that men do not come from great distances to dine well, or to attend brilliant entertainments. Unless the papers and discussions are worthy the occasion, the meeting will be a miserable failure, even though many thousands are in attendance and hospitality and good cheer are unlimited. The Congress meets for scientific purposes, and if these be not attained it meets in vain.

The foreign visitors who come to attend the meetings of Section XVII will be most warmly received. We know that we but express the hearty sentiments of every American dentist when we bid them an earnest welcome, and assure them that our hearts and our hands are as open to them as our doors. But they must not expect to find things precisely as at home. Americans are a practical people, and they give especial prominence to practical work. The meditative Englishman, the philosophizing German and the experimentalizing Frenchman will not find us indifferent to their methods, but they will see a prominence given to demonstration that is not witnessed at their homes. They will note, perhaps with surprise, that much of the time is given up to clinics and exhibition of appliances and methods, and the suspicion may enter their minds that too much attention is given to the mechanical. If we may with propriety speak of "American" dentistry—and when the term is used it of course simply means American methods of thought and practice, and does not imply a claim for any special system—it is essentially practical. We have not made such theoretical advance as have some of the older nations who are richer in true scientific lore than are we, but we are vain enough to believe that in operative work American dentists are the peers of any. A young and growing country has many mechanical obstacles to overcome which are unknown to nations that were old before America was discovered. This has developed the inventive and operative faculty in our people, and the dentistry of America partakes of our national characteristics. It will be our pride at the coming Congress to exhibit to the world the phases in which we believe we most excel. When our foreign brethren visit us then, let them not feel impatient at the prominence here given the actual, operative department of our profession, and we on our part will diligently strive to profit from their expositions of the theoretical, speculative, and more strictly scientific aspect, and a mutual respect and admiration with increased esteem will ensue.

